# Transmembrane Protein 208: A Novel ER-Localized Protein That Regulates Autophagy and ER Stress

**DOI:** 10.1371/journal.pone.0064228

**Published:** 2013-05-14

**Authors:** Yuanbo Zhao, Jia Hu, Guangyan Miao, Liujing Qu, Zhenda Wang, Ge Li, Ping Lv, Dalong Ma, Yingyu Chen

**Affiliations:** 1 Key Laboratory of Medical Immunology, Ministry of Health, Peking University Health Science Center, Beijing, China; 2 Center for Human Disease Genomics, Health Science Center, Peking University, Beijing, China; Istituto Nazionale per le Malattie Infettive, Italy

## Abstract

Autophagy and endoplasmic reticulum (ER) stress are both tightly regulated cellular processes that play central roles in various physiological and pathological conditions. Recent reports have indicated that ER stress is a potent inducer of autophagy. However, little is known about the underlying molecular link between the two processes. Here we report a novel human protein, transmembrane protein 208 (TMEM208) that can regulate both autophagy and ER stress. When overexpressed, TMEM208 impaired autophagy as characterized by the decrease of the accumulation of LC3-II, decreased degradation of autophagic substrates, and reduced expression of critical effectors and vital molecules of the ER stress and autophagy processes. In contrast, knockdown of the TMEM208 gene promoted autophagy, as demonstrated by the increase of LC3-II, increased degradation of autophagic substrates, and enhanced expression levels for genes key in the ER stress and autophagic processes. Taken together, our results reveal that this novel ER-located protein regulates both ER stress and autophagy, and represents a possible link between the two different cellular processes.

## Introduction

Autophagy is a highly conserved catabolic process involved in delivery of cytoplasmic components to lysosome for degradation [1], [2]. In normal nutrition condition, autophagy is maintained at a basic level to keep cellular homeostasis and can be induced when needed [1]. Deregulation of autophagy has been implicated in a wide range of pathologies, including cancer, myopathies, infections and neurodegenerative diseases [1].

Autophagy involves the sequestration of cytoplasmic components and intracellular organelles within a double-membrane vesicle, the autophagosome. Ultimately, the outer membrane of autophagosome fuses with the lysosome, and sequestered components are thereby delivered to the lysosome for degradation by lysosomal enzymes [2]. Two conserved protein conjugation systems are involved in the formation of autophagosome: the autophagy-related (ATG) ATG12-ATG5 conjugation systems and LC3 (a homologue of mammalian ATG8) [2]. ATG12-ATG5 conjugation is essential for the pre-autophagosome formation, and LC3-phosphatidylethanolamine (LC3-PE) modification is necessary for formation of autophagosome. During autophagy, LC3 is processed to cytosolic form (LC3-I) proteolytically, by C-terminal removal. LC3-I covalently links to phosphatidylethanolamine and is incorporated into autophagosome membranes where it recruits cargo. This lipidation process converts cytosolic LC3-I into the active, autophagosome membrane-bound form, LC3-II [2], [3]. LC3-II levels (compared with actin or tubulin loading controls) correlate with autophagosome numbers which reflect the level of cellular autophagy in a positive correlation [3]–[7]. Although autophagy has been considered a bulk degradation process of cellular materials with little or no selectivity, there is now evidence to support that the process may also show selectivity to its substrate [8]. Such examples of selective autophagy degradation have been reported for p62 [8], [9] and polyQ80 aggregates protein [10]. The levels of p62 and polyQ80 aggregates could be used to monitor autophagic flux in a negative correlation [4], [7]–[10]. Therefore, while molecules involved in the regulation of autophagy have been revealed [2], [11], many details regarding their cellular pathway remain unknown [12].

The endoplasmic reticulum (ER) is a cellular membrane compartment for secretion and membrane protein synthesis in eukaryotic cells, and an important initiation site for the formation of pre-autophagosome [12], [13]. Most nascent proteins are synthesized and able to fold to their native conformation in the ER. Unfolded or misfolded proteins however, trigger ER stress (also known as unfolded protein response) and are targeted to be degraded through a highly conserved, ER-associated quality control degradation system. Three ER transmembrane sensor molecules, PERK, ATF6 and IRE1, are involved in this process. Well-known downstream targets of these sensors include GRP78 and CHOP [13], [14]. In resting cells, the functions of the three ER stress sensors are inhibited by association with GRP78. Changes in physiological condition however, will cause GRP78 dissociation from these sensors and trigger ER stress [13]. ER stress has been implicated in many human diseases, such as neurodegenerative diseases, diabetes and heart disease [14], and hence understanding the underlying mechanisms involved in its molecular functions is of significant biological interest.

Recently studies have demonstrated that ER stress is a potent inducer of autophagy [15], [16]. However, the mechanisms by which the two cellular pathways connect to each other remain unknown. TMEM208 was firstly identified in a cDNA clone analysis and termed as HSPC171 [17]. The Mammalian Gene Collection (MGC) Program also identified the cDNA of TMEM208 [18]. However, the characteristics and function of this protein are unknown. Here we identified TMEM208, a highly conserved protein, is involved in both autophagy and ER stress. TMEM208 is an ER-located protein and was prone to forming SDS-resistant aggregates upon heat treatment. Overexpression of TMEM208 negatively regulated autophagy and ER stress, while gene knockdown of its expression enhanced autophagy and triggered ER stress. Our results implicate TMEM208 as a linker between ER stress and autophagy.

## Materials and Methods

### Antibodies and Reagents

A specific polyclonal antibody against TMEM208 was prepared by immunizing rabbits (Experimental Animal Center, Peking University Health Sciences Center, Beijing, China) with chemically synthesized TMEM208 peptides (KGKVGTRGKKQIFEENRET and EHNEKRQRRQERRQM). When the rabbit was ready for its post-immunization blood collection, it will be tranquilized with an intramuscular injection of a ketamine (8 mg/kg) -xylazine (1.6 mg/kg) cocktail and placed in a standard rabbit restraining box. All experimental procedures and protocols were approved by the Peking University Animal Ethics Committee. The collected rabbit serum was purified by peptide affinity chromatography via CNBr-activated Sepharose™ 4 Fast Flow (GE Healthcare), according to the manufacturer’s instructions. Other antibodies used in this study were: LC3B (Sigma Aldrich), ATG5 (MBL International), β-actin (Sigma Aldrich), FLAG (Sigma Aldrich), MYC (Tianjin Sungene), GRP78 (Bioworld Technology), CHOP (Cell Signaling Technology), GFP (Rockland), DyLight 680-conjugated secondary anti-mouse antibody (Rockland), DyLight 800-conjugated secondary anti-rabbit antibody (Rockland), FITC/TRITC-conjugated secondary antibodies against mouse or rabbit IgG (Jackson ImmunoResearch Inc.). Other reagents used in this study were: cDNA libraries of human normal adult tissues (Clontech), The Dual-Luciferase® Reporter (DLR™) Assay System (Promega), bafilomycin A_1_ (Sigma Aldrich), DTT (Sigma Aldrich) and Earle’s balanced salt solution (EBSS; Sigma Aldrich).

### Plasmids

The TMEM208 cDNA was amplified from cDNA library of U2OS cells and cloned into pcDNA3.1 vector (primers CCCAAGCTTATGGCGCCCAAGGGCAAA and CGGGATCCCTATAACCGCTTCATCTG). The following plasmids expressing truncated TMEM208 or tagged TMEM208 were also constructed: FLAG-TMEM208, TMEM208-myc, TMEM208-EGFP, TMEM208-TagRFP (tRFP), TMEM208_1–70_-EGFP, TMEM208_1–100_-EGFP, TMEM208_1-140_-EGFP, TMEM208_70–173_-EGFP, TMEM208_1–100_, TMEM208_1–140_ and TMEM208_70–173_. The plasmid for short hairpin RNA (shRNA) targeting TMEM208 was purchased from OriGene Technologies, Inc. polyQ19-luciferase and polyQ80-luciferase plasmids were gifts from Dr. Conrad C. Weihl [10]. Other plasmids utilized in this study were described previously [19]. All plasmids were confirmed by DNA sequencing.

### Cell Culture, Transfection and Treatment

U2OS and HEK293 cells were purchased from the Cell Bank of Chinese Academy of Sciences (Shanghai, China) and maintained in Dulbecco’s modified Eagle’s medium (DMEM) supplemented with 10% fetal bovine serum (FBS). U2OS and HEK293 have been used to study autophagy by several groups, including ours [10], [19]–[22]. GFP-LC3 stably expressing Hela cell line was from Dr. Wei-Guo Zhu [23]. Other cell lines used in this study were cultured routinely. Cells were transfected using Lipofectamine 2000 (Invitrogen) according to the manufacturer’s instruction. HEK293 cells stably expressing GFP-LC3 were obtained by prolonged selection with G418 after transfection [20]. Cell autophagy was induced by nutrient deprivation through incubation in Earle’s balanced salt solution (EBSS). The inhibition of autophagic flux was achieved by treating cells with 10 nM of bafilomycin A_1_, a vacuolar-type H^+^-ATPase inhibitor, which can block the fusion of autophagosomes with lysosomes. ER stress was induced by treating cells for 4 hours with 1 mM of DTT.

### RT-PCR and Quantitative Real-time PCR

Different tissues of cDNA libraries were constructed by PCR amplification with TMEM208-specific primers (5′-GTTCTACCTGCGGATCATACTG-3′ and 5′-AGCCAGAAGGACCAGACATAG-3′). For quantitative real-time PCR, total RNA was extracted from U2OS cells with TRIzol reagent (Invitrogen) and reverse transcribed into cDNA using the ThermoScript RT-PCR System (Invitrogen). Primers used were as follows: TCTACCTGCGGATCATACTGG and TAGCTGGCCCCATACACTG for TMEM208, and CAACTGTTACAATCAAGGTCTATGAAG and CAAAGGTGACTTCAATCTGTGG for GRP78. Other primer sequences utilized were from PrimerBank (http://pga.mgh.harvard.edu/primerbank/): ACCAAGGGAGAACCAGGAAACG and TCACCATTCGGTCAATCAGAGC for CHOP; CTGGATGAAGATTGGGATT and TGACCGAGGAGACGAGAC for ATF6; ATGACCGAAATGAGCTTCCTG and GCTGGAGAACCCATGAGGT for ATF4; AAGGCGCTTACAGCTCAATG and CTGGGAGGCATAGACCATGT for LC3B; AAAGATGTGCTTCGAGATGTGT and CACTTTGTCAGTTACCAACGTCA for ATG5; TAGAGCGAACACGAACCATCC and CACTGCCAAAACACTCATAGAGA for ATG12; and finally, GAAGGTGAAGGTCGGAGTC and GAAGATGGTGATGGGATTTC were used for GAPDH.

### Immunofluorescence, Fluorescence and Confocal Microscopy

U2OS cells were cultured in confocal dishes and treated as indicated, and either fixed with 4% paraformaldehyde and imaged, or permeabilized with 0.2% Triton X-100, followed by staining with the relevant antibody, before confocal imaging. For GFP-LC3 puncta observation, HEK293 and Hela cells stably expressing GFP-LC3 were transfected with the relevant plasmids (as indicated) and fixed, before the cells were observed by an Olympus FV1000 fluorescent microscope (Olympus, Japan).

### Western Blot Analysis

Western blot analysis was performed as previously described [19]. Whole cell extracts were obtained by directly lysing the cells with SDS sample buffer (Laemmli sample buffer). Cell lysates were incubated at 50°C for 30 minutes (to denature proteins), resolved by SDS-PAGE, transferred to nitrocellulose (NC) membrane (Whatman) and probed by indicated antibodies. The protein bands were visualized using DyLight 800-conjugated secondary anti-rabbit antibody, and the infrared fluorescence image was obtained using an Odyssey infrared imaging system (LI-COR Biosciences, USA).

### *In vitro* Luciferase Assay

U2OS cells were co-transfected with polyQ19-luciferase or polyQ80-luciferase plasmids and indicated constructs. Firefly luciferase activities were assessed by Veritas microplate luminometer (Turner Biosystems) according to the manufacturer’s protocol for the Dual-Luciferase® Reporter Assay System (Promega).

### Statistical Analysis

Data are expressed as the mean ± SD. Band intensity was quantified using ImageJ Software (NIH) and expressed as fold change relative to control. Statistical differences between groups were assessed using Student’s t-test, whereby *p*<0.05 was considered as statistical significance.

## Results

### Genetic Information and Expression Profile of Human TMEM208

**Figure 1:**
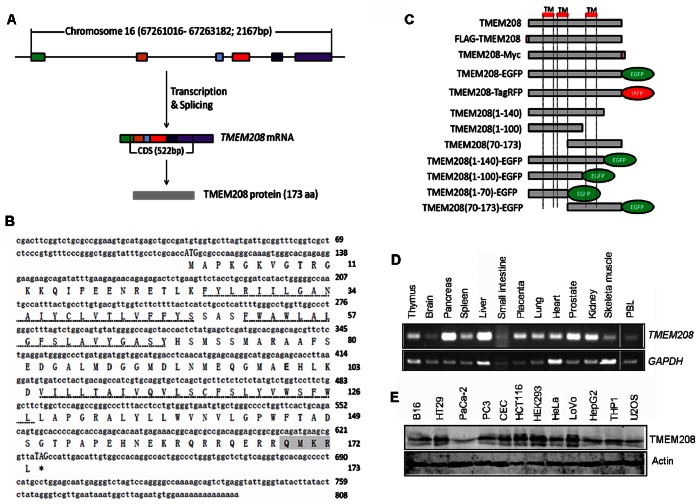
TMEM208 genetic information and expression profile. (A) Schematic of the gene and mRNA structure of *TMEM208*. The *TMEM208* gene is located on chromosome 16, has six exons, and encodes a protein with 173 amino acid residues. (B) Nucleotide and amino acid sequences for human TMEM208. The start and stop codons are upper case in the nucleotide sequence. The predicted TM domains are indicated with dashed lines. (C) Schematic representation of *TMEM208* constructs used in this study. (D) *TMEM208* mRNA expression was analyzed by RT-PCR in human normal tissues. *GAPDH* expression was amplified as an internal control. (E) Protein expression of TMEM208 in mammalian cell lines was detected by rabbit anti-TMEM208 antibody using Western blot. Actin was used as the loading control.

Human TMEM208 gene is located on chromosome 16q22.1 and encompasses six exons and five introns (Fig. 1A). The mRNA of TMEM208 is 808 base pairs long and encodes a predicted 19.6 kDa protein (173 amino acid residues) with an isoelectric point of 9.33. The full-length cDNA and amino acids are shown in Fig. 1B. Transmembrane (TM) prediction (http://www.cbs.dtu.dk/services/TMHMM/) suggests that TMEM208 has three potential transmembrane helices (Fig. 1B, dashed lines). The C-terminal is composed of QMKR, a KKxx-like motif (Fig. 1B, gray highlighted; predicted by http://psort.hgc.jp/form2.html). The KKxx motif is an endoplasmic reticulum (ER) membrane retention signal that exists in the C-terminal of many ER-located transmembrane proteins [24]. To our knowledge, no functional study has been performed on TMEM208.

The mRNA expression of TMEM208 was confirmed by RT-PCR in a variety of normal (non-cancerous) human tissue samples (Fig. 1D) that included thymus, brain, pancreas, spleen, liver, placenta, lung, heart, prostate, kidney, skeletal muscle and peripheral blood lymphocytes (PBL). Western blot analysis confirmed the presence of TMEM208 in several mammalian cell lines (Fig. 1E). TMEM208 was also widely expressed in many normal or tumor tissues obtained from immunohistochemistry (http://www.proteinatlas.org/ENSG00000168701).

Database searches using National Center for Biotechnology Information (NCBI; http://www.ncbi.nlm.nih.gov/) and European Bioinformatics Institute (EBI; http://www.ebi.ac.uk/) revealed that the TMEM208 sequence is highly conserved across many species, as demonstrated by multiple protein alignments (Fig. S1A). Evolution analysis also indicated that TMEM208 is evolutionarily highly conserved (Fig. S1C). We also found a highly conserved protein domain named DUF788 (Fig. S1B), whose function remains unknown. It should be noted that in all species compared in our studies, DUF788 domain covered almost the whole sequence of TMEM208.

### TMEM208 Protein is Prone to Aggregate upon Heat Treatment

**Figure 2:**
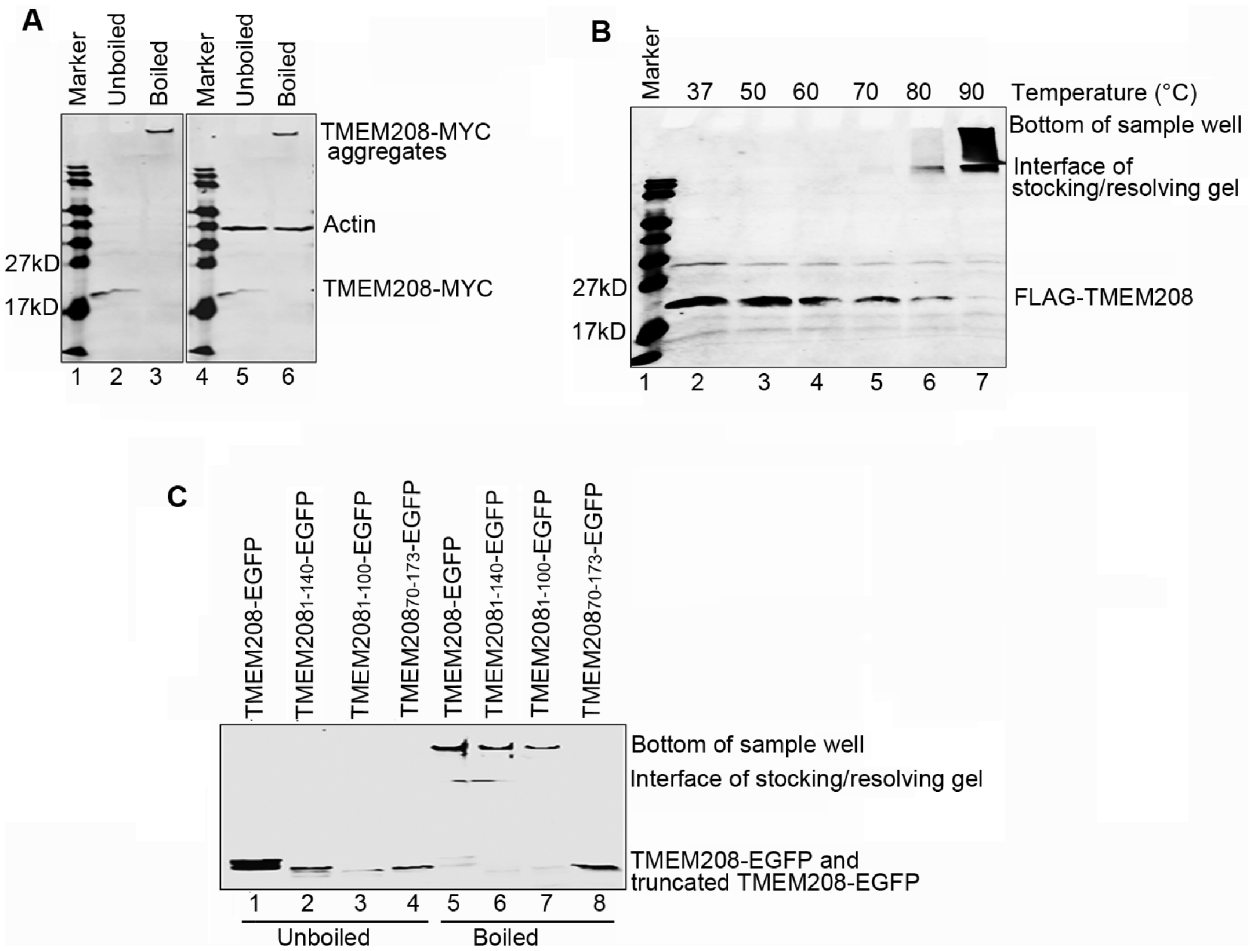
TMEM208 protein is prone to aggregation upon heat treatment. (A) U2OS cells were transfected with TMEM208-MYC or vector plasmids and harvested 24 hours post-transfection. Cell lysates were either boiled or left unboiled prior to Western blotting. TMEM208-MYC and β-actin were detected with anti-MYC and anti-β-actin antibodies, respectively. (B) U2OS cells were transfected with FLAG-TMEM208 for 24 hours. Cell lysates were loaded at different temperatures to aid protein denaturation prior to western blotting. FLAG-TMEM208 was detected with anti-FLAG antibody. (C) U2OS cells were transfected with truncated TMEM208-EGFP plasmids for 24 hours. Cell lysates were then boiled or left unboiled before Western blot. The expression of truncated TMEM208-EGFP was detected by anti-GFP antibody.

We unexpectedly discovered, during cell lysate denaturation in standard Western blot sample preparation, TMEM208 had a tendency to aggregate at high temperatures. TMEM208 proteins, when boiled, formed aggregates that precipitated on the NC membrane and did not migrate down the PAGE gel (Fig. 2A, left panel). The β-actin (control sample) was unaffected by the boiling treatment preparation (Fig. 2A, right panel). The formation of SDS-resistant aggregates has been reported for certain membrane proteins [25]. To determine the optimal denaturation temperature for TMEM208 protein, cell lysates were denatured in temperatures ranging from 37°C to 90°C. Above 50°C, the TMEM208 band present at the 19 kDa location gradually disappeared as the band at the top of the NC membrane gradually increased (Fig. 2B), which was indicative of aggregate formation.

Among the GFP-fusion constructs composed of truncated fragments of TMEM208 (Fig. 1C), cell lysates of TMEM208_1–100_-GFP and TMEM208_1–140_-GFP both formed aggregates (Fig. 2C) upon boiling. However, cell lysates from TMEM208_70–173_-GFP did not form SDS-resistant aggregates upon boiling (Fig. 2C). These results implicate that the N-terminal 70 amino acid residues of TMEM208, which contains two adjacent predicted transmembrane regions, are responsible for SDS-resistant aggregate formation.

### TMEM208 is Localized in the ER

**Figure 3:**
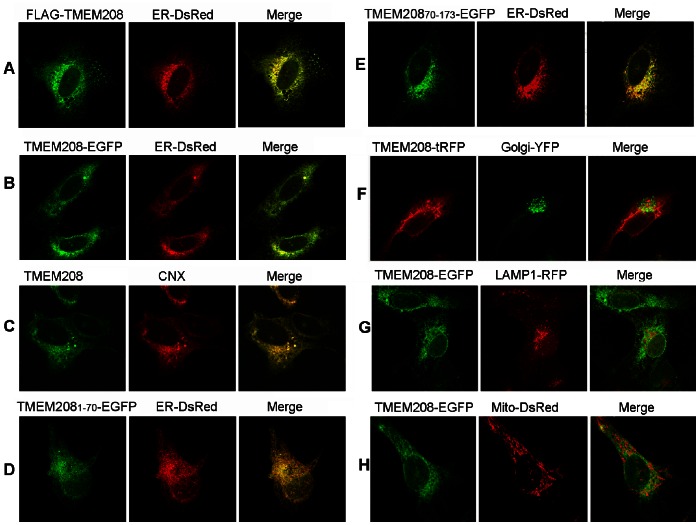
TMEM208 is localized in the ER. Confocal microscopy images of U2OS cells co-transfected with different plasmids: (A) FLAG-TMEM208 and ER-DsRed, immunostained with anti-FLAG antibody; (B) TMEM208-EGFP and ER-DsRed; (C) U2OS cells were immunostained with rabbit anti-TMEM208 and anti-calnexin (CNX); (D) TMEM208_1–70_-EGFP and ER-DsRed; (E) TMEM208_70–173_-EGFP and ER-DsRed; (F) TMEM208-tRFP and Golgi-YFP; (G)TMEM208-EGFP and LAMP1-RFP; (H) TMEM208-EGFP and Mito-DsRed. Images were obtained at 24 hours after transfection.

To determine the subcellular location of TMEM208, U2OS cells were co-transfected with FLAG-TMEM208 and ER-DsRed. The cells were then stained with FLAG antibody, and analyzed by confocal microscopy. Our results demonstrated that FLAG-TMEM208 co-localized with ER-DsRed (Fig. 3A). Co-transfection of TMEM208-EGFP with ER-DsRed also demonstrated this result (Fig. 3B). Endogenous TMEM208 and calnexin (CNX; an ER integral membrane protein) were also analyzed and their co-localization was confirmed by confocal microscopy (Fig. 3C). Taken together, the results suggest that TMEM208 is localized within the ER.

Interestingly, TMEM208_1–70_-EGFP and TMEM208_70–173_-EGFP were also found to be co-localized with ER-DsRed (Fig. 3D, E), which indicates that the C-terminal QMKR motif is not necessarily required for TMEM208 to be located within the ER. We also discovered that TMEM208 had no obvious co-localization with Golgi-DsRed (Fig. 3F), lysosome marker LAMP1-RFP (Fig. 3G) and the mitochondrial marker, Mito-DsRed (Fig. 3H), indicating that TMEM208 has an ER specific co-localization.

### TMEM208 Regulates Autophagy

**Figure 4:**
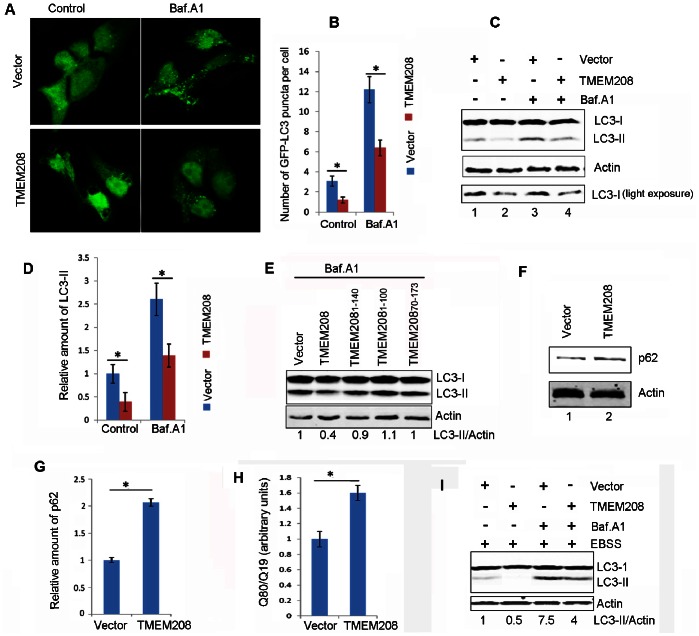
TMEM208 overexpression attenuates autophagy. (A) Confocal microscopy images of GFP-LC3 distribution obtained from HEK293 cells that stably expressed GFP-LC3, transfected with TMEM208 and vector plasmids for 24 hours. Bafilomycin A_1_ (Baf. A1, 10 nm) was added for the last 2 hours. (B) Quantification of GFP-LC3 puncta per cell, data are means ± SD of at least 100 cells scored (*P<0.05). (C) Western blot for endogenous LC3-II protein levels in U2OS cells treated as in (A). (D) Quantification of the amounts of LC3-II relative to β-actin treated as in (C). The average value in the vector-transfected cells without Baf. A_1_ treatment was normalized as 1. Data are the means ± SD of results from three experiments (*P<0.05). (E) U2OS cells were transfected with truncated TMEM208 plasmids for 24 hours, treated with Baf. A_1_ for the last 2 hours, before LC3-II protein levels were analyzed by Western blot. (F) Western blot for p62 protein levels in U2OS cells transfected with TMEM208 or vector plasmids for 24 hours. (G) Quantification of the amounts of p62 relative to β-actin treated as in (F). The average value in the vector-transfected cells was normalized as 1. Data are the means ± SD of results from three experiments (*P<0.05). (H) TMEM208 or vector plasmids were co-transfected with polyQ80-luciferase or polyQ19-luciferase plasmids, respectively. Luciferase activities were monitored at 30 hours post-transfection, and polyQ80-luciferase/polyQ19-luciferase ratios were calculated (*P<0.05). Degradation of polyQ19 was also monitored and served as an internal control. (I) U2OS cells were transfected with TMEM208 or vector plasmids for 24 hours, starved for 2 hours in the presence or absence of Baf. A_1_, before LC3-II protein levels were analyzed by Western blot.

We next investigate the biological activity of TMEM208. TMEM208 was overexpressed in HEK293 cells that stably express GFP-LC3. GFP-LC3 puncta were then monitored by fluorescent microscopy. As described in Fig. 4A, the overexpression of TMEM208 resulted in the reduced punctate distribution of GFP-LC3 when compared to control vector. Bafilomycin A_1_, a V-ATPase inhibitor, which can neutralize lysosomal pH and block the fusion of autophagosomes and lysosomes [4], [6], [7], was employed to monitor the autophagic flux. Although bafilomycin A_1_ (10 nM) treatment caused accumulation of the GFP-LC3 puncta in both TMEM208 and vector-transfected cells, but the abundance of GFP-LC3 dots in vector-transfected cells was greater than that in TMEM208-transfected cells (Fig. 4B). Similar results were also obtained in Hela cells stably expressing GFP-LC3 (Fig. S2), indicating that TMEM208 overexpression may impair autophagosome formation. We further analyzed the level of the endogenous membrane-bound LC3-phospholipid conjugate LC3-II by western blot. Compared with vector-transfected cells, the accumulation of LC3-II protein in TMEM208-transfected cells was much weaker in the presence and absence of bafilomycin A_1_ (Fig. 4C and D). Interestingly, only full length of TMEM208, and none of the truncated TMEM208-variants showed decreased accumulation of LC3-II (Fig. 4E). These data implied that the overexpression of TMEM208 impairs the autophagic flux under normal nutrient conditions. Consistent with our data suggesting that TMEM208 affected LC3-II synthesis, this treatment also led to an increase of the endogenous autophagic substrate p62 (Fig. 4F and G, lane 2 vs. lane 1). Likewise, overexpression of TMEM208 reduced the degradation of polyQ80 aggregates formed by a stretch of 80 glutamine residues that is also an autophagic substrate [4], [10] (Fig. 4H), indicating that TMEM208 overexpression decreases the clearance of autophagic substrates. Furthermore, under starvation conditions, TMEM208 overexpression also attenuated LC3-II accumulation with or without bafilomycin A_1_ treatment (Fig. 4I). Data from our repeated experiments indicates that TMEM208 overexpression could reduce cellular autophagic flux under rich nutrition and starvation conditions.

**Figure 5:**
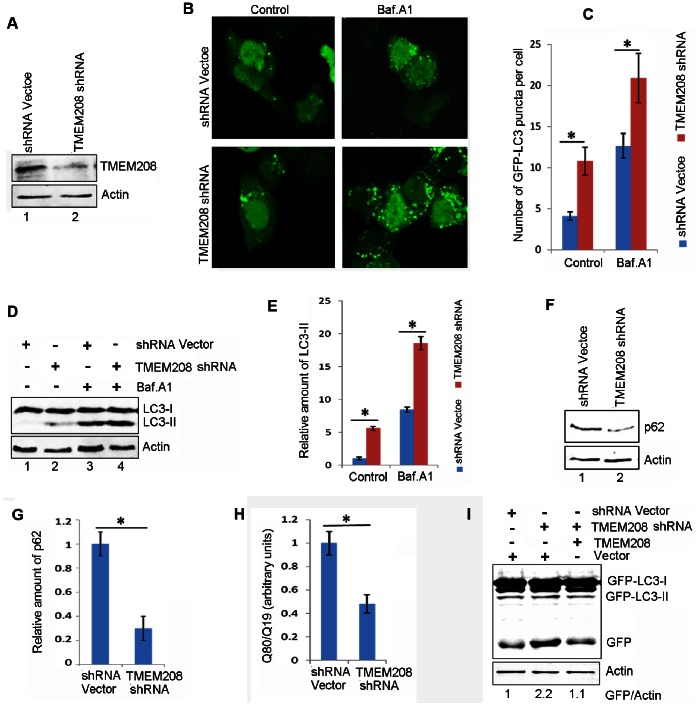
Knockdown of TMEM208 promotes autophagy. (A) U2OS cells were transfected with shRNA vector or shRNA targeting TMEM208 (TMEM208 shRNA). TMEM208 protein levels were detected by western blot at 72 hours after transfection. (B) Representative images of GFP-LC3 distribution in stable GFP-LC3 expressing HEK293 cells transfected with shRNA vector or TMEM208 shRNA for 72 hours, and treated with Baf. A_1_ (10 nM) for the last 2 hours. (C) Quantification of GFP-LC3 puncta per cell, data are means ± SD of at least 100 cells scored (*P<0.05). (D) Western blot analysis of endogenous LC3-II levels in U2OS cells treated as in (B). (E) Quantification of the amounts of LC3-II relative to β-actin treated as in (D). The average value in the vector-transfected cells without Baf. A_1_ treatment was normalized as 1. Data are the means ± SD of results from three experiments (*P<0.05). (F) Western blot analysis of p62 protein levels in U2OS cells transfected with shTMEM208 or shRNA vector plasmids for 72 hours. (G) Quantification of the amounts of p62 relative to β-actin treated as in (F). The average value in the shRNA vector transfected cells was normalized as 1. Data are the means ± SD of results from three experiments (*P<0.05). (H) U2OS cells were co-transfected with polyQ80-luciferase (or polyQ19-luciferase) and the indicated plasmids for 72 hours. PolyQ80-luciferase/polyQ19-luciferase ratios were analyzed using the Dual Luciferase Reporter System (*P<0.05). Degradation of polyQ19 was also monitored and served as an internal control. (I) Stable GFP-LC3 expressing HEK293 cells were co-transfected with shTMEM208 (or shRNA vector) and TMEM208 (or pcDNA3.1 vector) for 72 hours. GFP-LC3 and free-GFP levels were analyzed by Western blot.

To confirm the physiological role of TMEM208 in autophagy, further analysis was performed in TMEM208-silenced cells. The effectiveness of shRNA against TMEM208 was demonstrated by Western blot analysis (Fig. 5A). In HEK293 cells that stably expressed GFP-LC3, the occurrence of GFP-LC3 puncta was significantly increased in TMEM208-silenced cells when compared to shRNA-vector transfected cells (Fig. 5B and C). Likewise, bafilomycin A_1_ treatment resulted in a further increase of GFP-LC3 puncta in TMEM208 knockdown cells (Fig. 5B and C). Similar results were also observed in Hela cells stably expressing GFP-LC3 (Fig. S2), which suggests that the inhibition of TMEM208 expression enhances cellular autophagic flux. Consistent with these data, LC3-II levels were increased after knockdown of TMEM208 (Fig. 5D and E, lane 2 vs. lane 1). In Bafilomycin-A1-treated cells, TMEM208 knockdown further increased LC3-II levels (Fig. 5D and E, lane 4 vs. lane 3), suggesting that there was an induction of autophagosome synthesis. Additionally, these phenomena were accompanied with a decrease of the endogenous autophagic substrates p62 (Fig. 5F and G, lane 2 vs. lane 1) and an acceleration of polyQ80 degradation (Fig. 5H).

The specificity of TMEM208 shRNA was determined by a rescue experiment, whereby free GFP was used as a measure of cellular autophagic flux [4], and was shown to be more pronounced in TMEM208-knockdown cells (Fig. 5I, middle lane). Co-transfection of shRNA with the TMEM208 plasmid reversed the GFP levels such that they were similar to those obtained from control cells (Fig. 5I, right lane vs. left lane), indicating that cellular autophagy was increased by the inhibition of TMEM208 expression. Taken together, these observations confirm that TMEM208 could indeed negatively regulate cellular autophagy processes.

### TMEM208 Regulates ER Stress

**Figure 6:**
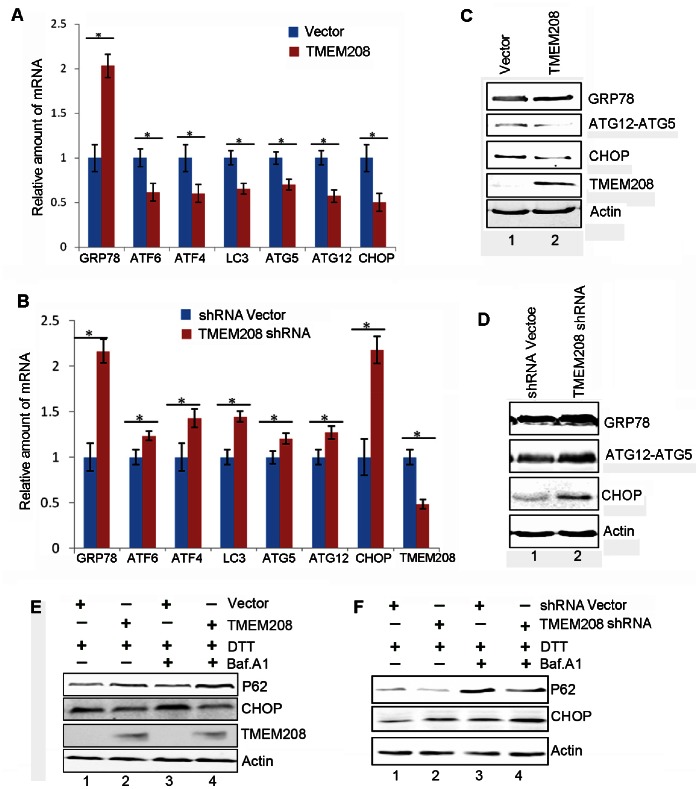
TMEM208 regulates ER stress. (A) U2OS cells were transfected with TMEM208 or pcDNA3.1 vector alone for 24 hours and cellular RNA were extracted for quantitative real-time PCR analysis of the genes indicated. (B) U2OS cells were transfected with shTMEM208 or shRNA vector for 72 hours, and relative mRNA levels of the indicated genes were analyzed by quantitative real-time PCR. The average value in the vector-transfected cells was normalized as 1. Data are the mean ± SD of results from three experiments (*P<0.05). (C and D) Western blot analysis of the protein levels of GRP78, ATG12-ATG5 conjugates and CHOP in U2OS cells treated as in (A) or treated as in (B). (E) U2OS cells were transfected with pcDNA3.1 vector or TMEM208 for 24 hours, then DTT (1 mM) and Baf. A1 were added to culture media for 4 hours as indicated. Cells were harvested and the levels of CHOP, P62 and TMEM208 were analyzed by western blot. (F) U2OS cells were transfected with shRNA vector or TMEM208 shRNA for 72 hours. DTT (1 mM) and Baf. A1 were added to culture media for the final 4 hours as indicated. Cells were harvested and the levels of CHOP and P62 were analyzed by western blot. Actin was detected as the protein loading control.

Considering that TMEM208 is located in the ER, we examined whether TMEM208 had any impact on ER stress. We established that overexpression of TMEM208 in U2OS cells resulted in the downregulation of mRNA levels for ER-stress marker molecules that included ATF6, ATF4 and CHOP (Fig. 6A). Downregulation of mRNA levels of key autophagy molecules, including LC3, ATG5 and ATG12 were also evident in TMEM208 overexpressing cells (Fig. 6A). In contrast, the knockdown of TMEM208 resulted in the upregulation of mRNA levels of GRP78, ATF6, ATF4, LC3, ATG5, CHOP and ATG12 (Fig. 6B). Interestingly, the levels of the ER-resident chaperone GRP78 mRNA, which is a classical marker and regulator of the ER stress response were significantly increased in both TMEM208 overexpressing and TMEM208 knockdown cells (Fig. 6A and B). Western blotting was used to confirm the protein levels of GRP78, ATG12-ATG5 and CHOP in both TMEM208-overexpressing and TMEM208-silenced cells (Fig. 6C and D).

To provide further support for our data suggesting that TMEM208 regulates ER stress, we analyzed the effect of TMEM208 in Dithiothreitol (DTT) treated U2OS cells using western blotting. DTT is commonly used to induce cellular ER stress [15], [26]. DTT-induced ER stress were indicated by the increased CHOP level (Fig. 6E and F, lane1), which is an ER stress marker [13], [14]. In the presence of DTT, TMEM208 overexpression decreased the levels of CHOP and led to an accumulation of p62 protein (Fig. 6E), indicating that TMEM208 activity impaired DTT-mediated ER stress and autophagy. When TMEM208 was depleted in U2OS cells, the cells displayed the enhanced the CHOP protein levels and increased the degradation of p62 protein (Fig. 6F), suggesting that inhibition of TMEM208 strengthened DTT-mediated ER stress and autophagy. Therefore, aforementioned data implicate that TMEM208 exerts a negative correlation on the modulation of ER-stress and autophagy.

## Discussion

Although the Human Genome Project (HGP) has been completed for almost ten years, the functions of many proteins encoded by human genome still remain unclear [27]. For example, while the molecular mechanisms of autophagy have been substantially studied, little is known about the regulation of autophagy at the ER level. In this study, we discover a novel ER-located transmembrane protein, TMEM208. TMEM208 is an evolutionarily conserved protein with a DUF788 domain of unknown function that spans almost the entire sequence. We successfully assigned TMEM208 function as a regulator and determined a possible link between ER stress and autophagy.

We propose that TMEM208 has three transmembrane (TM) domains, two of which are predicted to be at the N-terminal. In line with many other membrane proteins that contain significant secondary structure in the presence of SDS [25], we report that TMEM208 formed SDS-resistant aggregates at high temperatures. Furthermore, our investigations implicated the two adjacent N-terminal TM domains as the reason for aggregate formation upon heat treatment. In contrast, the C-terminal (70–173) of TMEM208, which contains one TM domain, failed to form aggregates (Fig. 2C).

Overexpression of TMEM208 impaired cellular autophagy process and ER stress, while knockdown of its expression promoted cellular autophagy and ER stress. Interestingly however, we discovered that the overexpression and knockdown of TMEM208 upregulated GRP78 expression (Fig. 6). GRP78 is a well-known ER molecular chaperone with a vital role in the regulation of ER stress [28]. GRP78 inhibits the ER stress pathway through binding to the ER-lumen regions of three essential ER stress sensors in non-stressed cells: PERK, ATF6 and IRE. Upon ER stress, GRP78 proteins dissociate from these sensor molecules and bind to the misfolded or unfolded proteins, activating the ER stress pathway [28]. Thus, high levels of GRP78 have an inhibitory effect on ER stress [26]. We propose that the overexpression of TMEM208 inhibits ER stress due to the upregulation of GRP78 expression. This was corroborated by our findings of reduced expression levels of other ER stress effectors, such as ATF4 and CHOP in TMEM208-transfected cells. The upregulation of GRP78 in our knockdown of TMEM208 may have resulted from cellular ER stress that triggered the ER stress pathway, enhancing the expression of ER-stress related molecules. Previous reports have implied that GRP78 expression is increased in ER stress as a result of ER stress signaling activation [13], [28]. Nonetheless, other mechanisms by which TMEM208 regulates ER stress cannot be excluded.

Collective data demonstrated that ER-stress related molecules have been indicated in the regulation of the expression of autophagy-related genes. Namely, ATF4 and CHOP can promote the expression of LC3 and ATG5, respectively, through binding to their promoters and enhancing their transcription [29], [30]. ATF4 can transcriptionally regulate the expression of ATG1/ULK1 [31]. ER-stress also upregulates ATG12 at both the mRNA and proteins levels and promote the conversion of LC3-I to LC3-II [32]. It is possible that TMEM208 regulates autophagy through the regulation of ER stress, i.e., TMEM208 inhibits ER stress, which results in the inhibition of expression of genes such as ATF4 and CHOP. In addition, the subsequent expression of essential ATGs is also inhibited. Considering that TMEM208 is located in the ER membrane, it is also possible that TMEM208 prevents autophagy through inhibiting the formation of the pre-autophagosome structure. However, the exact mechanism by which TMEM208 regulates autophagy warrants further investigation. Taken together, our findings provide new insights into the regulatory networks of autophagy and ER stress.

## Supporting Information

Figure S1 **TMEM208 is an evolutionarily conserved protein.** (A) Multiple protein alignments of TMEM208 in different species. (B) TMEM208 has a highly conserved protein domain, DUF788 (colored strips), shared by all species compared here. (C) Phylogenetic tree of TMEM208. Branch lengths are proportional to evolutionary distances. The units are the number of amino acid substitutions per site. The tree was generated using MEGA 5 software.
(TIF)

Figure S2 **TMEM208 regulates autophagy in HeLa cells.** Hela cells stably expressing GFP-LC3 were transfected with indicated plasmids for 48 hours, and then observed by confocal microscopy.
(TIF)
