# Supplementary figures and images for: Transmembrane Protein 208: A Novel ER-Localized Protein That Regulates Autophagy and ER Stress
Source: PLoS One. 2013 May 14;8(5):e64228. doi: 10.1371/journal.pone.0064228 (PMC3653875; doi:10.1371/journal.pone.0064228)

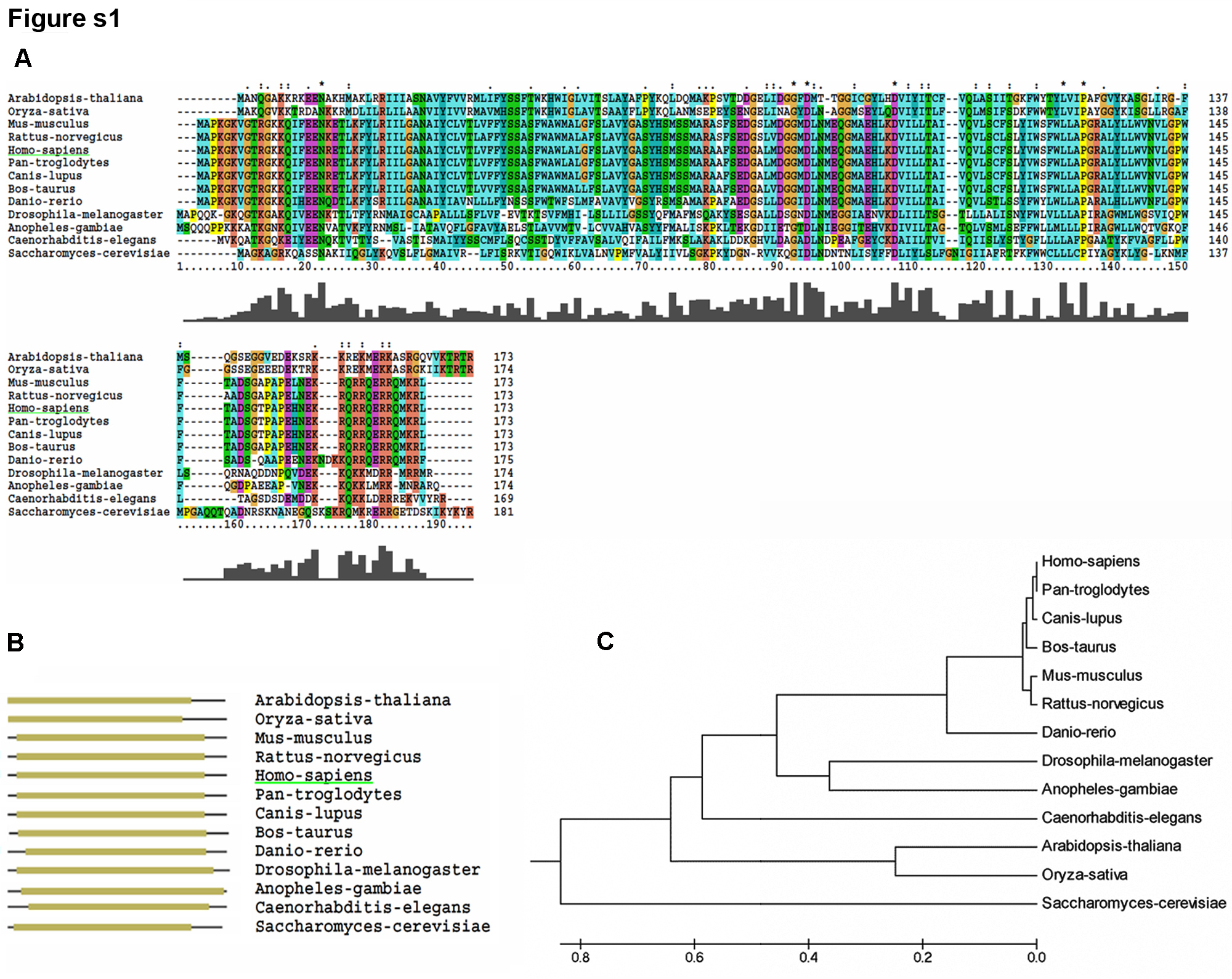

Supplement: Figure S1 — TMEM208 is an evolutionarily conserved protein. (A) Multiple protein alignments of TMEM208 in different species. (B) TMEM208 has a highly conserved protein domain, DUF788 (colored strips), shared by all species compared here. (C) Phylogenetic tree of TMEM208. Branch lengths are proportional to evolutionary distances. The units are the number of amino acid substitutions per site. The tree was generated using MEGA 5 software. (TIF) [file pone.0064228.s001.tif]

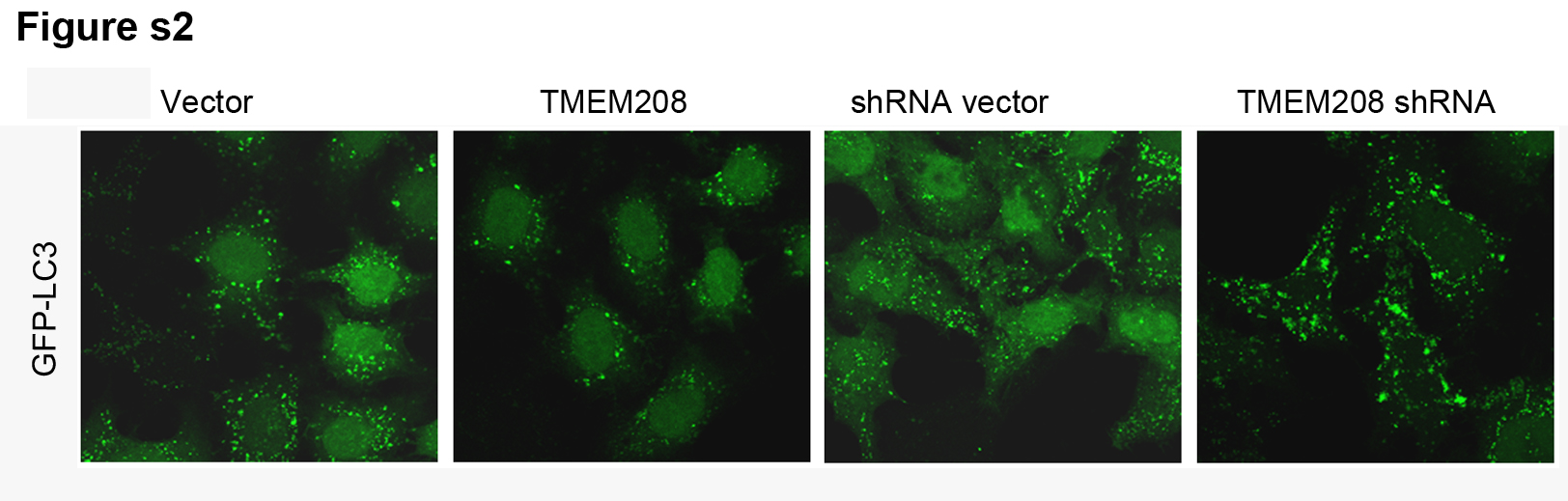

Supplement: Figure S2 — TMEM208 regulates autophagy in HeLa cells. Hela cells stably expressing GFP-LC3 were transfected with indicated plasmids for 48 hours, and then observed by confocal microscopy. (TIF) [file pone.0064228.s002.tif]
